# Adipose tissue supports normalization of macrophage and liver lipid handling in obesity reversal

**DOI:** 10.1530/JOE-17-0007

**Published:** 2017-03-30

**Authors:** Maayan Vatarescu, Sapir Bechor, Yulia Haim, Tal Pecht, Tanya Tarnovscki, Noa Slutsky, Ori Nov, Hagit Shapiro, Avishai Shemesh, Angel Porgador, Nava Bashan, Assaf Rudich

**Affiliations:** 1 The Department of Clinical Biochemistry and PharmacologyFaculty of Health Sciences, Ben-Gurion University of the Negev, Beer-Sheva, Israel; 2 The National Institute of Biotechnology in the Negev (NIBN)Ben-Gurion University, Beer-Sheva, Israel; 3 The Shraga Segal Department of MicrobiologyImmunology and Genetics, Ben-Gurion University, Beer-Sheva, Israel

**Keywords:** adipose tissue macrophages, obesity reversal, adipose tissue inflammation, glucose production, insulin resistance

## Abstract

Adipose tissue inflammation and dysfunction are considered central in the pathogenesis of obesity-related dysmetabolism, but their role in the rapid metabolic recovery upon obesity reversal is less well defined. We hypothesized that changes in adipose tissue endocrine and paracrine mechanisms may support the rapid improvement of obesity-induced impairment in cellular lipid handling. C57Bl-6J mice were fed *ad libitum* either normal chow (NC) or high-fat diet (HFF) for 10 weeks. A dietary obesity reversal group was fed HFF for 8 weeks and then switched to NC for 2 weeks (HFF→NC). Whole-body glucose homeostasis rapidly nearly normalized in the HFF→NC mice (fasting glucose and insulin fully normalized, glucose and insulin tolerance tests reversed 82% to the NC group levels). During 2 weeks of the dietary reversal, the liver was significantly cleared from ectopic fat, and functionally, glucose production from pyruvate, alanine or fructose was normalized. In contrast, adipose tissue inflammation (macrophage infiltration and polarization) largely remained as in HFF, though obesity-induced adipose tissue macrophage lipid accumulation decreased by ~50%, and adipose tissue MAP kinase hyperactivation was reversed. *Ex vivo*, mild changes in adipose tissue adipocytokine secretion profile were noted. These corresponded to partial or full reversal of the excess cellular lipid droplet accumulation induced by HFF adipose tissue conditioned media in hepatoma or macrophage cells, respectively. We propose that early after initiating reversal of nutritional obesity, rapid metabolic normalization largely precedes resolution of adipose tissue inflammation. Nevertheless, we demonstrate a hitherto unrecognized contribution of adipose tissue to the rapid improvement in lipid handling by the liver and by macrophages.

## Introduction

Adipose tissue inflammation is frequently viewed as a central mechanism in the pathogenesis of obesity-associated glucose intolerance and metabolic dysregulation. In humans, association studies demonstrated that adipose tissue inflammation (particularly macrophage infiltration) characterizes obese persons who develop insulin resistance (Harman-Boehm *et al*. 2007), but to a much lesser degree age, sex and BMI-matched obese persons who remain insulin sensitive (Kloting *et al*. 2010). In mouse models amenable to detailed time-course analyses, even short-term (3 days) high-fat feeding (HFF) induced neutrophil-dominated adipose tissue inflammation. This inflammatory response clearly occurred before the development of obesity (Elgazar-Carmon *et al*. 2008, Talukdar *et al*. 2012), but already could be tied to insulin resistance, particularly in the liver (Elgazar-Carmon *et al*. 2008, Hadad *et al*. 2013).

The secretory profile of adipose tissue is thought to be altered by adipose tissue inflammation and mediate its impact on whole-body metabolic state. Auto-paracrine communication affects the function of cells within adipose tissue, and distant tissues (muscle, brain and pancreas) are endocrine targets of secreted products from adipose tissues. Indeed, the particularly strong association of visceral adiposity with insulin resistance, glucose intolerance and dyslipidemia could also implicate adipose tissue inflammation, as visceral fat exhibits higher inflammatory markers compared to subcutaneous fat (Harman-Boehm *et al*. 2007, Item & Konrad 2012). Moreover, the direct communication of visceral fat with the liver via the portal vein implicates the liver as a central target for the endocrine dysfunction caused by visceral fat inflammation (Rytka *et al*. 2011, Item & Konrad 2012). Consistently, various means of interfering with obesity-related adipose tissue changes, including inhibition of inflammatory cascades and the stress-related MAP kinase JNK, resulted in decreased hepatic steatosis, insulin resistance and glucose overproduction (Sabio *et al*. 2008, Wueest *et al*. 2010, Zhang *et al*. 2011).

Compared to this wealth of studies implicating adipose tissue inflammation in the *pathogenesis* of obesity-related metabolic dysfunction, much less is known about its role in the *resolution* of glucose intolerance and impaired lipid handling upon caloric restriction and/or obesity reversal. A striking, and poorly explained observation, in both humans and experimental models, has been that dysglycemia can be very rapidly normalized after bariatric surgery (Varela 2011, Bradley *et al*. 2012). Changes in incretins, bile acids, possibly engaging the microbiota, have been raised as possible mechanisms (Cho 2014). Clearly, resolution of obesity itself is not required for this metabolic improvement, which in some patients even results in remission of preoperative overt type 2 diabetes. Moreover, when inducing weight loss in HFF mice, dysglycemia improved without apparent resolution of adipose tissue inflammation (Jung *et al*. 2013), and lipid droplets in adipose tissue macrophages (ATM) were even increased early in the course of caloric restriction (Kosteli *et al*. 2010). Yet, reversal of obesity-associated liver changes (steatosis) may be better aligned with metabolic normalization (Fabbrini *et al*. 2009). Thus, it remains largely unknown whether resolution of obesity-associated glucose and lipid dysmetabolism is largely independent of adipose tissue or whether normalization of the paracrine/endocrine function of adipose tissue remains a contributing factor, despite grossly retained adipose tissue inflammation.

Here, we challenged the hypothesis that early in the course of reversal of diet-induced obesity, adipose tissue’s paracrine and endocrine function contributes to metabolic normalization, in particular, of lipid handling within adipose tissue and by hepatocytes. Furthermore, we assessed whether these functions associate with the reversal of obesity-induced adipose tissue inflammation, stress response and increased ATM lipid content.

## Methods

### Reagents

Tissue culture medium, serum and antibiotic solutions were obtained from Biological Industries (Beit-HaEmek, Israel). Recombinant human insulin was from Novo Nordisk. Source and catalogue numbers for all antibodies and PCR primers are presented in Supplementary Table 1 (see section on supplementary data given at the end of this article).

### Animals and treatments

The study was approved in advance by Ben-Gurion University Institutional Animal Care and Use Committee and was conducted according to the Israeli Animal Welfare Act following the guidelines of the Guide for Care and Use of Laboratory Animals (National Research Council 1996). Six-week-old male C57BL/6J mice (Harlan Laboratories, Rehovot, Israel) were acclimatized for 2 weeks, housed 2–4/cage in a monitored animal facility with 12:12 light:darkness cycle. Mice had free access to filtered water and standard rodent chow (11% calories from fat, 65% from carbohydrates and 24% from protein; Altromin, Lage, Germany). At the age of 8 weeks, mice were randomly divided into 3 groups for a 10-week dietary intervention: (i) normal chow (NC, as detailed previously); (ii) high-fat diet (HFF, 58.7% calories derived from fat, 25.5% carbohydrate and 15% protein, D12492; Research Diets, New Brunswick, NJ); (iii) reversibility group (HFF→NC) was given HFF for 8 weeks followed by 2 additional weeks of NC (all *ad libitum*). The two-week time point was chosen to serve our hypothesis focusing on early liver and adipose tissue changes at a time with significant but incomplete body weight reversal, but a near-complete normalization of glycemic control. Mice were weighed weekly at the same hour. For insulin signaling, *in vivo* insulin was injected after overnight fast 12 min before the mice were killed with CO_2_ or isoflurane. Tissue lysates were prepared in RIPA lysis buffer from 15 mg liver or 100 mg epididymal adipose tissue, as previously detailed (Nov *et al*. 2013). When not displayed in the graph, number of mice/group for each analysis is mentioned in the legends and was always derived from 2 to 4 independent experiments.

### Insulin and HOMA-IR measurements

Glucose was measured using a glucometer (Abbott). Insulin was measured in serum by an ultra-sensitive ELISA kit (Crystal Chem Inc., Downers Grove, IL, USA, catalogue #90800). HOMA-IR (homeostatic model assessment-insulin resistance) was calculated as fasting glucose (mg/dL) × fasting insulin (mIU/L)/405.

### Glucose, insulin, pyruvate, alanine and fructose tolerance tests

Mice were fasted for 4 h for the insulin tolerance test (ITT, 0.2 U insulin/kg body weight), and overnight for all other loading tests (glucose, alanine, pyruvate or fructose, all at 2 g/kg). Blood was drawn from the tail at the indicated time points. Area under the glucose excursion curve (AUC) was calculated by GraphPad software (Prism 5).

### Histology

Dissected liver and adipose tissue were fixed in 4% (v/v) paraformaldehyde at 4°C, processed and embedded in paraffin. Sections of 5 µm were stained with hematoxylin and eosin (H&E). Images were taken using an Olympus BX60 microscope. The percentage of liver histological sections area containing lipid-laden heptaocytes was estimated in 4 different X20 fields, 4 mice per group, from H&E-stained sections using an in-house MATLAB-based software. Adipocyte cell-size distribution was assessed as we previously reported (Nov *et al*. 2013).

### FACS analysis of adipose tissue stromal-vascular fraction (SVF)

It was performed after collagenase digestion and an additional wash with 10 mM EDTA to ensure the separation of lipid-laden macrophages, as we previously described (Nov *et al*. 2013, Shapiro *et al*. 2013). After FcBlock (BD Biosciences, Franklin Lakes, NJ, USA), cells were stained with the following conjugated antibodies (10 min on ice in the darkness): CD45-APC, F4/80-PE-Cy7 (both from E-Bioscience, San Diego, CA, USA) and CD11b-APC-Cy7 (BD Pharmingen, San Diego, CA, USA). Cells were washed and pellets were then stained for 20 min on ice with BODIPY 493/503 (3 μg/mL BODIPY for 5 × 10^6^ cells; Invitrogen, D3922). Stained samples were further washed and filtered using 100 μm mesh. Propidium iodide (0.2 μg/mL; Sigma, P4864) was added to all samples. Stained samples were analyzed by FACS (Canto, BD Biosciences).

### Tissue culture

Hepa-1c mouse hepatoma cells and RAW264.7 mouse macrophage cell line (American Type Culture Collection, Manassas, VA, USA), were maintained in Dulbecco’s Modified Eagle Medium (DMEM), 4.5 g/L glucose containing 10% (v/v) FBS, 50 U/mL penicillin, 50 µg/mL streptomycin and 4 mM glutamine (Biological Industries Ltd). For co-culture experiments, Hepa-1c cells were grown to 90% confluence. Lipid acumulation assays in 3 × 10^4^ Hepa-1c cells/well and 2.5 × 10^4^ RAW264.7 cells/well were plated in 96-well uClear, black plates (Greiner Bio One, Kremsmünster, Austria). All cells were at passage number below 20.

### Co-culture and conditioned media preparation

Epididymal fat pads from NC, HFF or HFF→NC were minced into 2–3 mm^3^ fragments (100 ± 5 mg/mL) and incubated in full medium for 24 h for relaxation, as we previously described (Nov *et al*. 2013, Slutsky *et al*. 2016). The pads were washed with sterile phosphate-buffered saline, and then either co-cultured with Hepa-1c cells for additional 24 h (100 mg tissue/well) or incubated in fresh medium that was collected (conditioned media, CM). Some HFF fat pads were treated after 2-h relaxation period with 20 μM SP600125 (JNK inhibitor) and 20 μM SB203580 (p38 inhibitor, both from Sigma-Aldrich) for 24 h in full serum medium (Supplementary Figure 1, see section on supplementary data given at the end of this article). The pads were washed thoroughly with sterile PBS and co-cultured with Hepa-1c cells for additional 24 h. At the end of the co-culture/conditioned media period, Hepa-1c cells were rinsed and serum starved (DMEM + 0.5% (w/v) BSA) for 3 h, after which explants and media were removed, cells were rinsed and stimulated with 100 nM insulin for 7 min. Cells were then rinsed twice with ice cold sterile PBS and then frozen at −80°C until further analysis by Western blot, as we previously described (Slutsky *et al*. 2016).

### Lipid accumulation

Hepa-1c and RAW264.7 cells were treated for 6 h with AT-CM (20% or 50% (v/v), respectively, diluted in DMEM). BODIPY and Hoechst 33342 (Invitrogen) were added (to a final concentration of 0.2 µg/mL and 1 µg/mL, respectively), and cells were imaged in a fully automated and unbiased manner using X40 wide-angle lens-equipped microscope (Operetta, PerkinElmer) (37°C, 5% CO_2_). The number of lipid droplets per cell was calculated by the image analysis software Columbus (Perkin Elmer). Each condition was examined in triplicate, and at least 27 fields per well were captured for statistical analysis.

### RNA extraction and quantitative RT-PCR

Total RNA from fat pads was extracted with the RNeasy lipid tissue mini kit (Qiagen) and analyzed with a NanoDrop. RNA (200 ng) were reverse transcribed with High Capacity cDNA Reverse Transcriptase Kit (Applied Biosystems). We used TaqMan system probes (Applied Biosystems) for real-time PCR amplification. Relative gene expression was obtained after normalization to *Rplp0 (36b4)* and *Hprt* using the formula 2^−ΔΔCT^.

### Adipokine array

To screen for putative AT-derived mediators, we used an antibody-based protein array (Proteome Profiler: Mouse adipokine array, R&D Systems, ARRY013) according to the manufacturer’s instructions (Slutsky *et al*. 2016). Quantification was performed using a transmission-mode scanner and image analysis software. Average signal of pixel density from duplicate spots/adipokine was determined using GelQuant NET software (Ver. 1.7.8, University of California, San Francisco, USA). The relative intensity of the reference values (three inside control duplicates in each membrane) was included in the densitometry calculations.

### Lipolysis assay

Adipose tissue CM (100 mg tissue/mL) from the 3 groups was used for free fatty acids (FFA) measurement (NEFA-HR kit, Wako Diagnostics), according to the manufacturer’s instructions.

### Statistical analysis

Data are expressed as the mean ± s.e.m., and calculations were performed using GraphPad Software (Ver. Prism 5 and Prism 6). Percent reversibility of all parameters was calculated as (HFF-(HFF→NC))/(HFF-NC) × 100. Unless otherwise stated, statistically significant differences between 2 groups were evaluated using the Student’s *t* test, whereas 3 or more groups were assessed by ANOVA followed by Tukey *post hoc* analysis for the between-group comparison.

## Results

**Figure 1:**
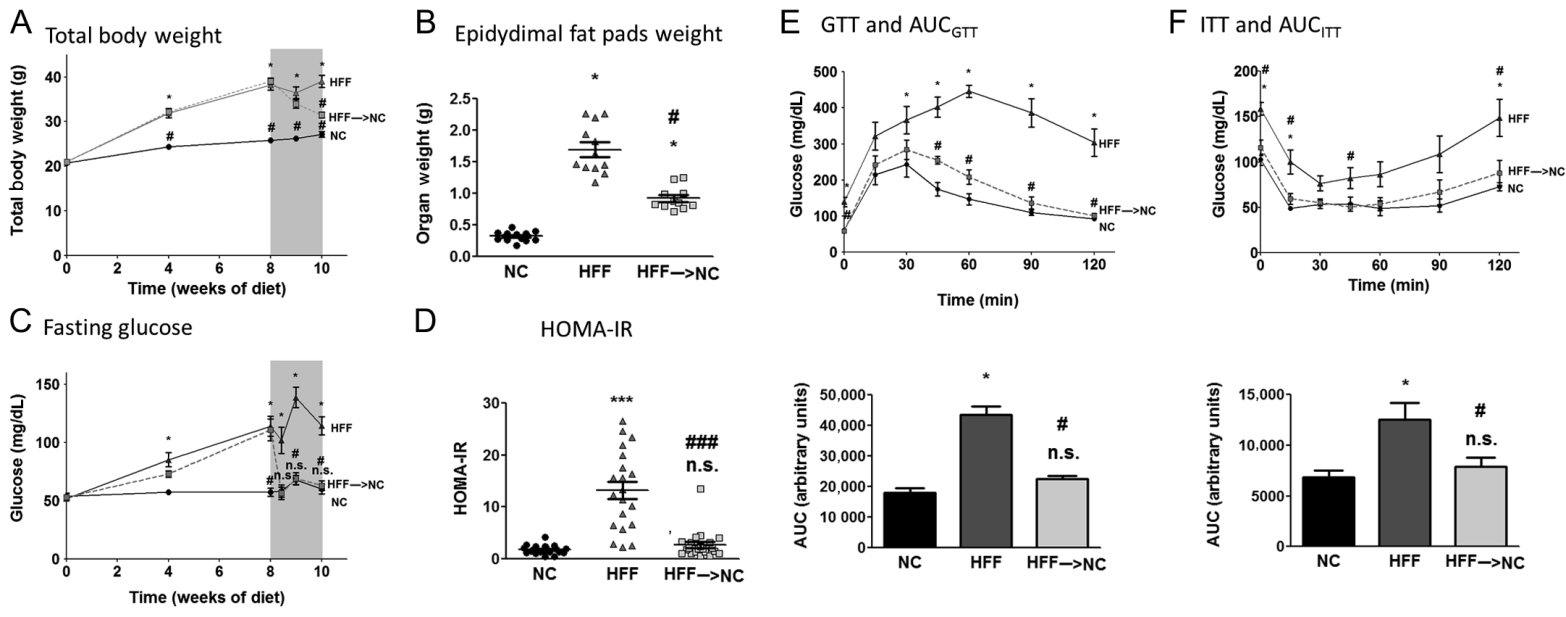
Glucose homeostasis and insulin sensitivity upon dietary switch. Eight-week-old C57BL6 mice were fed normal-chow diet (NC, ~10% kcal from fat) for 10 weeks, high-fat feeding (HFF, ~60% kcal from fat) for 10 weeks or HFF for 8 weeks and then switched to NC for 2 weeks (‘reverse group’, HFF→NC). Total body weight (BW) dynamics (A), epididymal fat at 10 weeks (B), circulating fasting blood glucose (C) and the glucose-to-insulin ratio (homeostatic model of insulin resistance – HOMA-IR) index 7 days after dietary reversal (D) are shown. *n* = 16–21 per group. Dynamically, intra-peritoneal glucose tolerance test (i.p. GTT, E) and intra-peritoneal insulin tolerance test (i.p. ITT, F) were performed 7 days after the dietary switch by i.p. administration of 2 g/kg BW glucose after an overnight fast or 0.2 U/kg BW insulin after 4-h fast, respectively. Area under the curves (AUC) of the GTT and ITT were calculated as well. For (E) *n* = 8/group, (F) *n* = 5–8/group. *P < 0.05 compared to NC; ^#^*P* < 0.05 compared to HFF; ***P < 0.001 compared to NC; ^###^*P* < 0.001 compared to HFF; n.s. – non-significant (*P* > 0.05) compared to NC.

To determine the early dynamics of recovery from obesity-associated metabolic dysfunction, we utilized the high-fat-fed (HFF) mouse model. All mice were fed *ad libitum*, either a normal chow (NC) diet, or HFF, for 10 weeks. The dietary reversal group was switched back to NC after 8 weeks on HFF and followed for the subsequent two weeks (HFF→NC). Within two weeks of the dietary reversal, mice lost ~60% of their excess total body and epididymal fat mass (‘excess’ being the difference between the HFF and the NC groups, Fig. 1A and B). Strikingly, fasting glucose levels normalized to the NC group levels 3 days after the dietary switch (Fig. 1C), and by the end of one-week follow-up, fasting-state insulin resistance, as determined by the HOMA-IR index, was fully reversed (Fig. 1D). Using dynamic testing of glucose and insulin tolerance demonstrated that within a week, HFF→NC mice were statistically indistinguishable from the NC control group in their glucose values in most time points (Fig. 1E and F). The area under the glucose tolerance test (GTT) or the insulin tolerance test (ITT) curves revealed 82% reversal of the difference between the obese HFF and the lean control, NC mice. Additional serum biochemical parameters of the mice are presented in Supplementary Table 2. Jointly, results demonstrate a rapid, nearly complete, reversal of insulin resistance and glucose intolerance induced by nutritional obesity upon switching back from obesogenic to normal diet.

**Figure 2:**
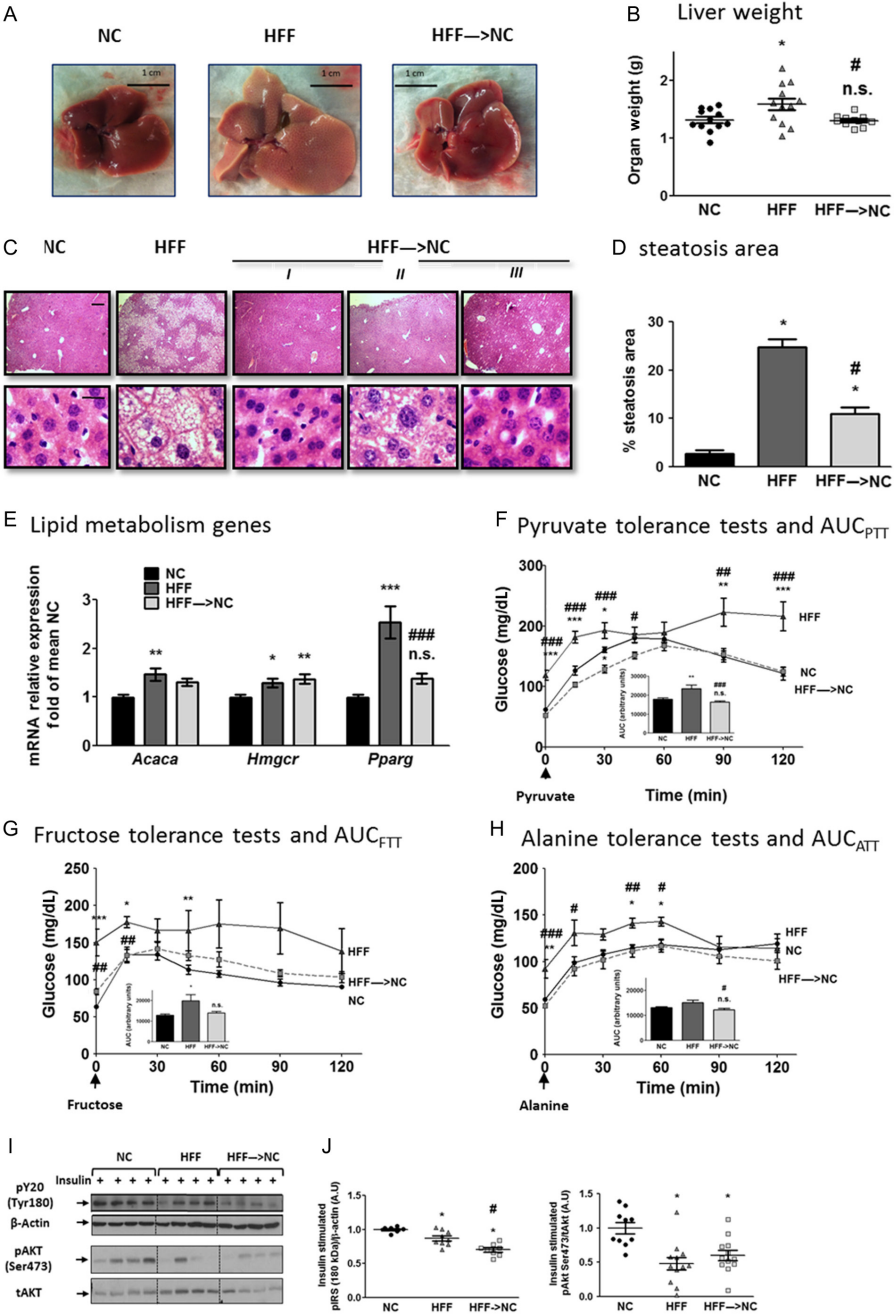
Early hepatic response to obesity reversal. Representative images (A) and weights (B) of livers 14 days after dietary switch (*n* = 11–12/group). (C) Representative liver hematoxylin and eosin (H&E)-stained histological sections of the three groups. *I*, *II* and *III* are sections from 3 individual mice from the HFF→NC group, demonstrating the variability in this group. (D) Mean area identified as steatotic (*n* = 4 for NC, HFF and *n* = 8 for HFF→NC. (E) Quantitative real-time PCR of liver expression of lipid metabolism genes: acetyl CoA carboxylase1 (*Acaca*), HNG CoA reductase (*Hmgcr*) and peroxisome proliferator-activated receptor γ (*PPARγ*). Values are the ΔΔCT values adjusted to Rplp0 and Hprt and are presented as fold of mean NC values (*n* = 15–16/group, from 4 independent experiments). (F, G and H) Results of pyruvate, fructose and alanine tolerance tests (PTT, FTT and ATT, respectively): twelve days after dietary reversal, mice were injected intraperitoneally with 2 g/kg BW each of the respective metabolites after an overnight fast. Glucose excursions during the following 2 h are shown. Insets are graphs of the calculated area under the curve (AUC). *n* = 9–10/group for PTT, *n* = 5–6/group for FTT and ATT). (I and J) Representative blots and densitometry of insulin signaling molecules in the liver. Mice were fasted overnight, and killed 12 min after intraperitoneal injection of 0.2 U/kg insulin. *n* = 7–12/group, from 2 to 3 independent experiments. *P < 0.05 compared to NC; ^#^*P* < 0.05 compared to HFF; ** or ^##^*P* < 0.01 compared to NC or HFF, respectively; *** or ^###^*P* < 0.001 compared to NC or HFF, respectively; n.s. – non-significant (*P* > 0.05) compared to NC.

Largely consistent with the robust reversal of HFF-induced changes within 2 weeks of dietary switch were the liver findings. Ten weeks of HFF resulted in significant excess liver weight, histologically explained by marked steatosis and hepatocyte ballooning (Fig. 2A B, C and D). Liver weights of the HFF→NC group were identical to the NC group, and histologically, a marked reversal of hepatic steatosis was observed. mRNA levels of either the rate-limiting enzyme of de-novo lipogenesis (acetyl CoA carboxylase, *Acaca*) or of cholesterol biosynthesis (HMG-CoA reductase, *Hmgcr*) and the lipogenic transcription factor PPARγ (*Pparg*), increased in HFF mice compared to those of NC (Fig. 2E). The effect of HFF on Pparg expression was completely abrogated in the HFF→NC group.

Hepatic steatosis is tightly associated with enhanced glucose production, contributing to dysglycemia. To determine the functional impact of the rapid loss in liver fat and its contribution to the near-normalization of whole-body glycemia, we assessed whole-body glucose production by loading tests with different gluconeogenic substrates. HFF mice exhibited significant glucose overproduction from pyruvate, fructose or alanine, compared to NC mice (Fig. 2F, G and H). The glucose excursion curves in the HFF→NC group were mostly indistinguishable from the NC control group for all three substrates, and the area under the curves showed a tendency for glucose production to be ‘over normalized’ when pyruvate or alanine were used as gluconeogenic substrates (Fig. 2F, G and H, insets). We did not detect significant changes in gluconeogenic enzymes’ mRNA levels, though glucose-6-phosphatase (*G6pc*) mRNA tended to be ~30% lower in HFF→NC mice compared to HFF mice (data not shown). Interestingly, hepatic insulin signaling responsiveness, as assessed by acutely administering a supraphysiological insulin bolus and measuring insulin-stimulated phosphorylation events by Western blotting, demonstrated insulin resistance in the HFF compared to the NC mice’ livers. Yet, this hepatic signaling defect was not reversed in the HFF→NC group (Fig. 2I). Collectively, the rapid normalization of glucose tolerance and insulin sensitivity upon dietary switch was largely matched by robust reversal of liver steatosis and whole-body glucose overproduction, though not of the impaired hepatic insulin signaling hyporesponsiveness.

**Figure 3:**
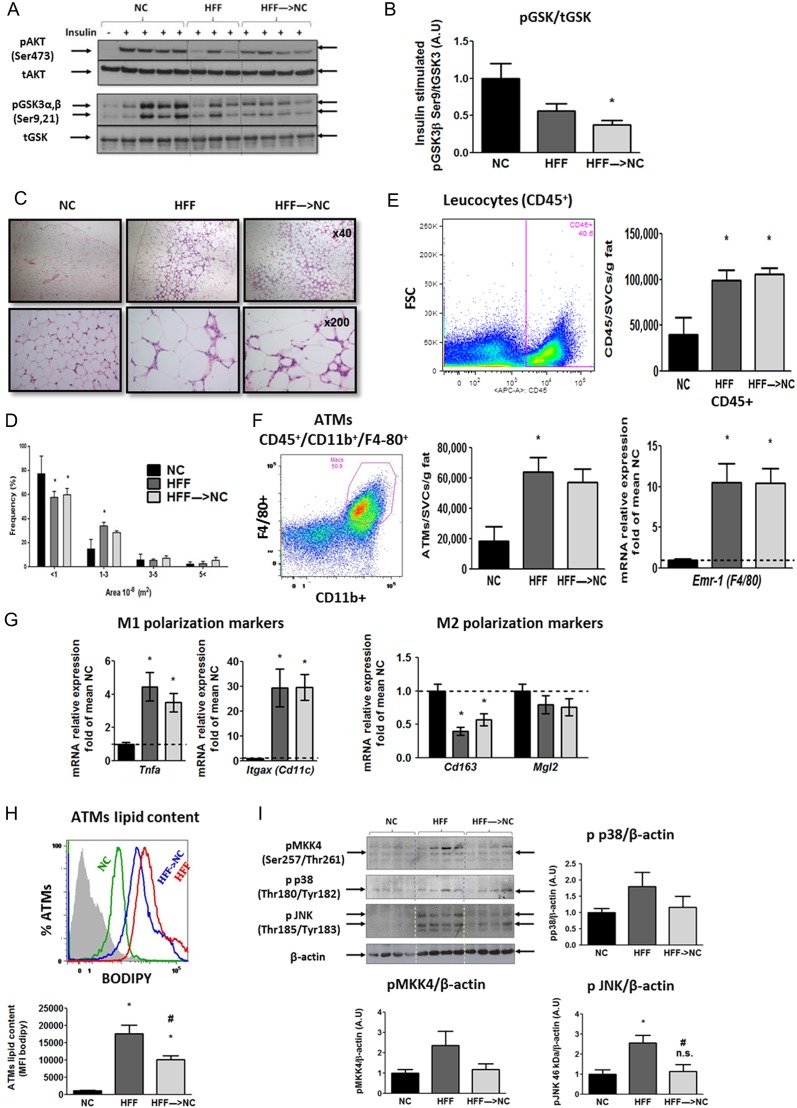
Adipose tissue adaptation to 2 weeks obesity reversal. (A and B) Representative blots and densitometry (*n* = 7–12/group from 2 to 3 independent experiments) of insulin signaling proteins in epididymal adipose tissue of mice treated as detailed in Fig. 2I and J. (C and D) Representative adipose tissue histological sections stained with H&E, and estimated size distribution. Flow Cytometry analyses of adipose tissue stromal-vascular cell fraction: Leucocytes were identified as CD45^+^ (E), adipose tissue macrophages (ATMs) as CD11b^+^F4/80^+^ (F), and ATM lipid content was assessed using the neutral lipid fluorescent dye BODIPY (H). Results are mean ± s.e.m. of mice from 2 independent experiments. Quantitative real-time PCR analysis in epididymal adipose tissue: *Emr-1* (F4/80) as a marker for macrophages (F), *Tnfa* was used as a classical pro-inflammatory cytokine and with *Itgax* (Cd11c) as M1 markers, and *Cd163* and *Mgl2* as markers of alternatively activated, M2 macrophages (G). Values are the ΔΔCT adjusted to *Rplp0* and *Hprt*, and are represented as fold of mean value of the NC group (*n* = 14–17/group from 5 independent experiments). (I) Representative blots and densitometry of adipose tissue stress signaling proteins. Densitometry are mean ± s.e.m. of *n* = 11–16/group from 3 to 4 independent experiments, with the mean value of the NC group defined as a value of 1. n.s. or *P < 0.05 vs NC, ^#^*P* < 0.05 vs HFF. A full colour version of this figure is available at http://dx.doi.org/10.1530/JOE-17-0007.

Given the importance of the fat-liver axis in the pathogenesis of obesity-associated dysmetabolism, we next assessed if the rapid near-normalization of glucose handling, insulin resistance and liver changes were also apparent at the adipose tissue level. We focused on adipose tissue insulin signaling, inflammation as well as MAP kinase stress signaling and ATM lipid content, all of which were implicated in the pathogenesis of obesity-related whole-body and liver metabolic dysfunction. As seen in the liver (Fig. 2I), 10-weeks HFF resulted in impaired insulin-stimulated phosphorylation of key insulin signaling kinases in the adipose tissue, and this impairment was not improved when mice were switched to NC in the last 2 weeks (Fig. 3A and B). Histologically, compared to NC, adipose tissue of HFF mice exhibited significant adipocyte hypertrophy (Fig. 3C), which was still evident with only minor decrease in estimated medium-sized cells in HFF→NC mice (Fig. 3D). In addition, multiple typical crown-like structures in the HFF mice’ adipose tissue seemed similarly abundant in the HFF→NC mice (Fig. 3C). Consistently, analysis of the stromal-vascular cell fraction of adipose tissue (obtained after collagenase digestion) by FACS revealed that the number of CD45^+^ leucocytes was increased in the HFF compared to the NC mice and remained similarly increased even after 2 weeks of dietary reversal (Fig. 3E). ATMs were identified from the CD45^+^ cell fraction by being double-positive for CD11b and F4/80 (Fig. 3F). Here, too, a similar increase was observed in the HFF and the HFF→NC groups compared to that in the NC control mice. This was further supported by whole adipose tissue mRNA content of *Emr-*1 (F4/80), which was markedly elevated in both HFF and HFF→NC compared to NC control (Fig. 3F).

To further estimate the impact of 2-week dietary reversal on adipose tissue inflammation, we assessed the mRNA levels of ATM M1/M2 polarization markers. Whole adipose tissue *Tnfa* levels were elevated 3.8- to 4.2-fold in both the HFF and HFF→NC mice compared to those in NC (Fig. 3G). The classical M1 polarization (and dendritic cell) marker CD11c was elevated 30-fold in HFF over NC and remained similarly elevated in the HFF→NC mice. Correspondingly, the ATM M2 polarization markers, *Cd163* and *Mgl2*, were attenuated by HFF, and were only slightly, and not significantly, reversed by the 2-week dietary reversal (Fig. 3G). Thus, obesity-related adipose tissue inflammation, at least as evidenced by adipose tissue ATM infiltration and polarization state, was not reversed by the 2-week dietary switch.

Lipid-laden ATMs, or adipose tissue foam cells, have been associated with metabolic dysfunction in obesity (Shapiro *et al*. 2013). Furthermore, they were demonstrated to induce adipose tissue insulin resistance (Shapiro *et al*. 2013). To determine if ATM lipid content was affected by the 2-week dietary switch, we utilized the neutral lipid fluorescent dye BODIPY (Fig. 3H). HFF markedly increased BODIPY mean fluorescence intensity in ATM compared to that in NC. HFF→NC mice exhibited a ~45% lower ATM lipid content than HFF. This finding suggests that despite seemingly maintained adipose tissue inflammation and macrophage number and polarization state, ATM lipid content, a signature of their lipid mishandling in obesity that potentially contributes to adipose tissue dysfunction, was significantly reversed.

An activated adipose tissue MAP kinase signaling cascade in human adipose tissue, consisting of the MAP3K ASK1 (MAP3K5), MKK4,3 and 6 and p38 MAP kinase and JNK, was shown to link obesity with whole-body insulin resistance (Rudich *et al*. 2007). Consistently, HFF induced increased activation (dual-phosphorylation) of p38 MAP kinase and JNK and their upstream kinase MKK4 (Fig. 3I). These changes were reversed in the HFF→NC group. Collectively, insulin responsiveness and common indicators of adipose tissue inflammation were not revered in HFF→NC. Nevertheless, increased ATM lipid content and adipose tissue MAP kinase activation were significantly decreased. These two adipose tissue parameters demonstrate rapidly-reversible obesity-related features of adipose tissue, which associate with the improved whole-body metabolism during obesity reversal.

**Figure 4:**
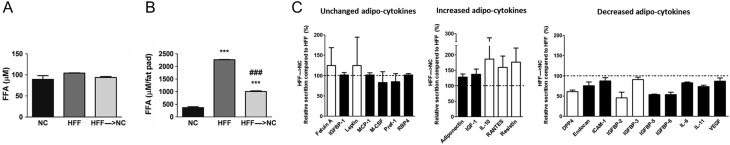
Changes in adipocytokine secretion from adipose tissue upon dietary switch. (A) FFA concentrations in CM of adipose tissue (100 mg tissue/mL medium). (B) Total FFA production by epididymal fat pads. (C) Adipocytokine array was used to screen for changes induced by dietary switch in the secretion of adipocytokines by cultured adipose tissue fragments. The levels of each adipocytokine were quantified by densitometry and expressed as fold of HFF for each adipokine. The detected factors were divided to those which qualitatively changed similarly (black bars) or differently (white bars) to their change induced in HFF adipose tissue by incubation with p38MAPK + JNK inhibitors (results of this analysis is presented in Supplementary Fig. 1). Results are mean ± s.d. of 2–3 independent experiments.

To challenge the hypothesis that auto-paracrine and/or endocrine mechanisms might contribute to the early metabolic improvement during obesity reversal, we screened for potential changes in secreted products from adipose tissue of HFF→NC vs HFF mice. Adipose tissue-conditioned media were prepared using a fixed tissue mass/volume ratio (100 mg tissue per 1 mL media), and secreted products from this tissue were analyzed. The concentration of free fatty acids (FFA) was not different between media of adipose tissue of HFF vs HFF→NC mice (Fig. 4A). Yet, when considering the adipose tissue weight, total delivery of FFA from the epididymal fat pad was increased in HFF and was markedly decreased in HFF→NC (Fig. 4B), demonstrating that the dysregulated basal lipolytic activity of this fat depot in obesity was in fact reversed by dietary reversal. Next, conditioned media of adipose tissue explants was screened by an adipocytokine array, revealing highly diverse response of these secreted products in adipose tissue of HFF→NC compared to HFF (Fig. 4C). Among others, concentrations of leptin and RBP4 were not different in HFF→NC compared to HFF, adiponectin and IL-10, but also resistin – were increased, and IL-6 and DPP4 decreased (Fig. 4C). To estimate if the decrease in activation of p38 MAPK and JNK (Fig. 3I) could account for these changes in the secretion of adipokines, we compared these changes to those induced by incubating adipose tissue from HFF mice with inhibitors of these two kinases (Supplementary Fig. 1). Qualitatively, 13 of 22 proteins (59%) exhibited similar changes (marked by the black bars, Fig. 4C). Most notable were the increase in adiponectin and decreases in Endocan, ICAM-1, IGFBP5 and 6, IL-6, 11, Pref-1 (DLK1) and VEGF.

**Figure 5:**
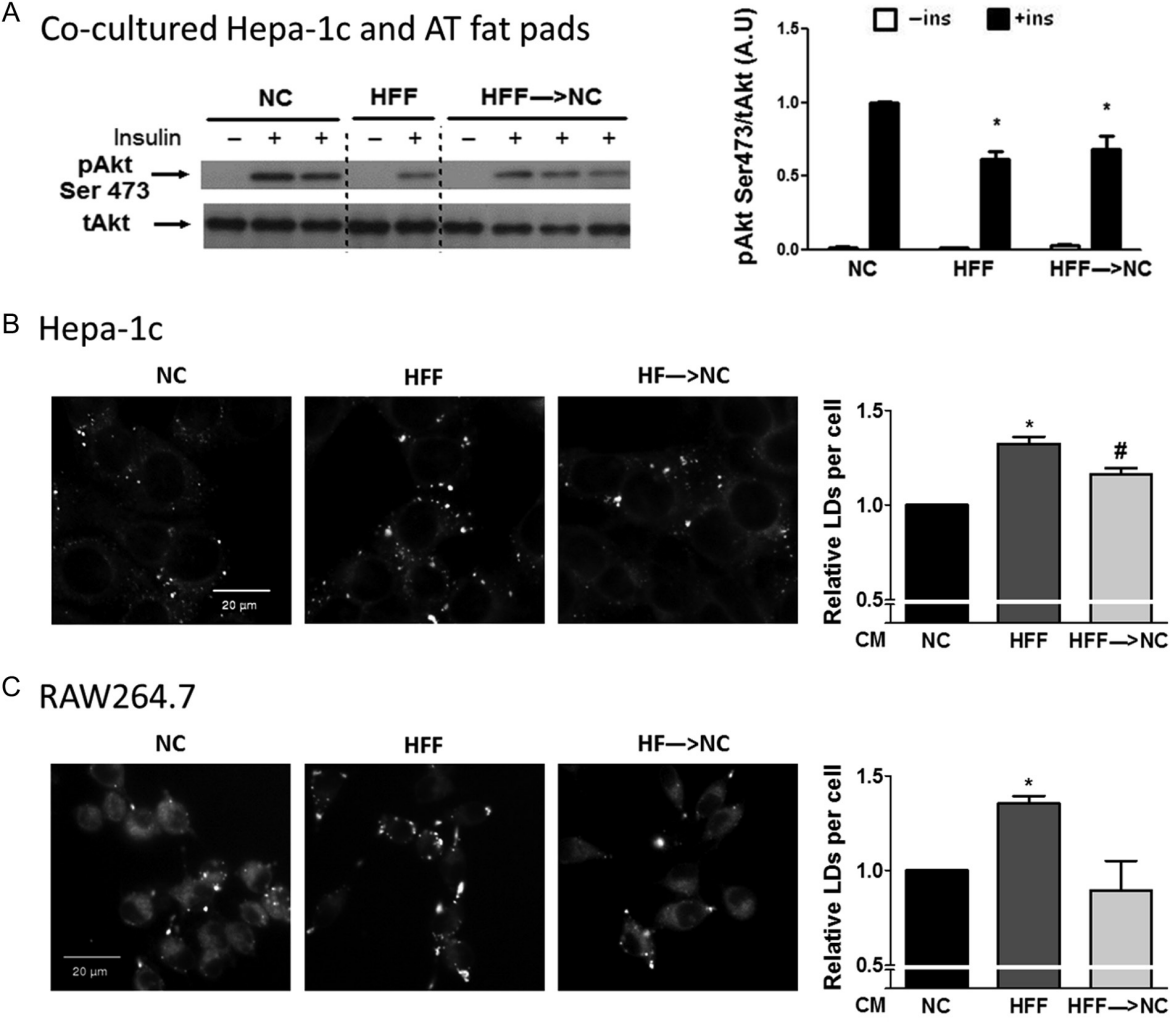
Functional reversal of increased liver cell and macrophage lipid storage by adipose tissue conditioned medium. (A) Insulin-stimulated Akt phosphorylation in Hepa-1c hepatoma cells exposed to conditioned media of adipose tissue from NC, HFF, and HFF→NC mice. Shown is a representative blot and densitometry analyses normalized to the signal in insulin stimulated cells pre-treated with conditioned media of NC mice. *N* = 4–20 individual wells from 2 independent experiments, *P < 0.05 vs NC. Hepa-1c hepatoma cells (B) and RAW264.7 macrophages (C) were treated with adipose tissue conditioned media (AT-CM) for 6 h. Lipid droplets (LDs) were stained with BODIPY 493/503 neutral lipid dye and imaged by Operetta high content imaging system. Graphs represent the relative number of LDs per cell, compared to NC treated cells. The results are expressed as mean ± s.e.m. of five (Hepa-1c) or four (RAW264.7) independent experiments, each preformed in triplicates. * or ^#^*P* < 0.05 compared to NC or HFF, respectively.

Finally, we wished to assess the overall impact of the observed changes in secretory profile induced by dietary reversal on adipose tissue’s endocrine communication (with liver-derived cells) and auto-paracrine communication (i.e., with macrophages). Hepatoma cells co-cultured with adipose tissue of HFF exhibited impaired insulin responsiveness compared to NC, and this impairment was also apparent upon exposure to adipose tissue from HFF→NC adipose tissue (Fig. 5A). This largely recapitulated insulin signaling *in vivo* (Fig. 2I), which was not improved in the HFF→NC group compared to HFF and remained lower than that in NC. For assessing the potential impact of adipose tissue on lipid accumulation by liver-derived cells, conditioned media of HFF adipose tissue was used. Higher lipid droplet formation was observed in liver-derived cells incubated with condition media of HFF adipose tissue compared to that of NC, an effect that was significantly less pronounced (though not normalized) in cells exposed to HFF→NC media (Fig. 5B). Addressing auto-paracrine actions was performed by assessing macrophage lipid accumulation as ATM in HFF→NC mice had lower lipid content than HFF (Fig. 3H). RAW264.7 macrophages incubated with conditioned media of HFF adipose tissue had higher lipid content than those exposed to NC adipose tissue media, and this was not apparent with conditioned media from the HFF→NC mice (Fig. 5C). This finding supports a role for improved paracrine communication within adipose tissue in mice early after dietary reversal of obesity.

## Discussion

The results of this study demonstrate that despite continued macrophage infiltration and M1 polarization in adipose tissue, *functionally*, adipose tissue’s auto-paracrine and endocrine communication contributes to normalization of lipid handling by macrophages and liver-derived cells early upon dietary reversal of obesity: lipid accumulation in macrophages was fully normalized when incubated with CM from HFF→NC mice, and partial reversal of the enhanced lipid accumulation by liver-derived cells was also observed. Secretion of selected adipocytokines, including increase in adiponectin and IL-10 and decrease in IL-6 and RBP4 by adipose tissue explants were associated with this effect, which could be partially recapitulated by incubating adipose tissue from HFF mice with MAP kinase inhibitors. These results from the *ex vivo* systems nicely reflected the early response to dietary reversal observed *in vivo* in obese mice: Like in the *in vitro* system, in the HFF→NC mice, liver insulin signaling after administering an insulin bolus (i.e., hepatic insulin responsiveness) was also not improved, consistent with previous studies (Jung *et al*. 2013). Nevertheless, in adipose tissue, ATM lipid content was decreased, suggesting a reversal of the contribution of adipose tissue foam cells to dysmetabolism. Moreover, hepatic steatosis and the accompanying increased whole-body glucose production were significantly/fully normalized. Jointly, our results highlight a measurable contribution of adipose tissue to the rapid metabolic normalization upon dietary reversal of obesity via auto-paracrine and endocrine mechanisms. Notably, greater changes in adipose tissue inflammation may further contribute to metabolic normalization after more extended periods of obesity reversal (Kosteli *et al*. 2010), beyond the single time point studied herein.

A central role is now assigned to adipose tissue in the pathogenesis of obesity-associated metabolic dysfunction, with particular emphasis on the fat-liver axis (i.e., in hepatic steatosis, insulin resistance and glucose overproduction) (Item & Konrad 2012, Kloting & Bluher 2014). Thus, the robust reversal of the hepatic steatosis and glucose overproduction in the HFF→NC group is somewhat surprising given the relatively minor reversal of adipose tissue changes after 2 weeks, including many common markers of adipose tissue inflammation. This may suggest that despite the central role of adipose tissue in obesity development, other tissues such as muscle (not assessed herein), or the liver, predominate early in the reversal phase. Indeed, in the rapid glycemic improvement seen after bariatric surgery, incretins and bile acids have been raised as potential major mediators (Laferrere 2011, Noel *et al*. 2016). These highlight a gut-liver axis that may predominate over fat-liver communication, and indeed, liver-autonomous mechanisms (or at least mechanisms unrelated to adipose tissue) are likely to play a role. Yet, using *ex vivo* systems that can isolate specific inter-organ/cell-type interactions from the multiple changes occurring *in vivo*, our data suggest that functionally there is a potential contribution of adipose tissue, via auto/paracrine and endocrine mechanisms. This could engage various mediators, including FFA, protein or steroidal hormones and other lipid mediators, secreted directly to the extracellular environment and/or, as recently proposed, via exosomes (Lazar *et al*. 2016). The total delivery of FFA to the liver (i.e., when considering both the secretion and the mass of the adipose tissue) is markedly decreased in HFF→NC compared to HFF, and thus, likely plays a role in decreasing lipid accumulation in both ATM and the liver. Yet, the *ex vivo* system utilized herein demonstrated effects beyond FFA as the concentration of either glycerol or FFA in the media that was generated by a wt/vol ratio were not different, likely because 100 mg of HFF tissue includes a smaller number of adipocytes than the same mass of NC or HFF→NC adipose tissue.

Screening for secreted adipocytokines (i.e., proteins), our data suggest that some factors are not reversed in the HFF→NC group, whereas others do change, including increase in adiponectin and IL-10 and decreases in adipose tissue secretion of DPP4 and IL-6. Increased adiponectin has been shown to mediate improved fat-liver communication when adipose tissue of HFF mice was treated with a lysosomal/autophagosomal inhibitor (Slutsky *et al*. 2016), though such improved insulin signaling was not observed here. Nevertheless, adiponectin is causally linked to obesity-related steatosis by knockout and transgenic models (Kim *et al*. 2007, Liu *et al*. 2012), the latter demonstrating that overexpression of adiponectin can prevent liver steatosis despite marked obesity. A very recent study in mice suggested that increased adiponectin and expression of its receptors in the liver mediate the advantageous combination of caloric restriction (dietary switch) and exercise, over each intervention alone (Cho *et al*. 2016). Decreased circulating IL-10 was recently shown to associate with the severity of NAFLD (Paredes-Turrubiarte *et al*. 2016), and treatment with a PEGylated IL-10 was shown to decrease liver lipid content, possibly acting on Kupffer cells (Chan *et al*. 2016). Interestingly, adiponectin suppresses macrophage lipid accumulation (Tian *et al*. 2012, Wang *et al*. 2013) and increases IL-10 production (Park *et al*. 2007). This provides a putative intra-adipose tissue, adipocyte–macrophage paracrine communication, leading to beneficial adipose–liver endocrine communication, which ultimately supports the rapid metabolic response to dietary switch. Adipose tissue release of DPP4 was shown to strongly associate with visceral fat and clinical characteristics of dysmetabolic obesity (Sell *et al*. 2013), and DPP4 inhibitors seem to prevent and/or improve liver steatosis (Rohrborn *et al*. 2015). Although local production of DPP4 in the liver may be the most biologically relevant source for NAFLD (Miyazaki *et al*. 2012), decreased delivery of adipose-derived DPP4 to the liver could also contribute, possibly interacting with higher incretin delivery during obesity reversal. Finally, IL-6 is likely the cytokine most implicated in obesity-related NAFLD development and progression. In particular, in obesity, adipocyte JNK activation was tied in mice to NAFLD by regulating IL-6 as an endocrine mediator in the fat-liver axis (Sabio *et al*. 2008). Indeed, comparing changes in the adipocytokine profile of HFF→NC vs HFF to the effect of combined pharmacological inhibition of adipose tissue JNK and p38 MAP kinase suggests the following pathway: obesity reversal rapidly normalizes p38 MAPK and JNK hyperactivation, resulting in changes in adipocytokine secretion from adipose tissue (including increases in adiponectin and IL-10 and decreased secretion of DPP4 and IL-6). These contribute to the improvement in hepatic steatosis and dysglycemia. Indeed, the potential clinical significance of obesity-associated hyperactivation of an ASK1-MKK4-p38/JNK MAP kinase signaling cascade in visceral fat was previously reported (Bashan *et al*. 2007, Bluher *et al*. 2009).

The auto-paracrine impact of obesity reversal within adipose tissue could be demonstrated by the finding that CM from HFF→NC adipose tissue completely normalizes the enhanced macrophage lipid accumulation induced by CM from HFF mice. *In vivo*, although adipose tissue leucocytes and macrophages, as well as their polarization markers, were not significantly reversed within 2 weeks of dietary switch, ATM lipid content decreased by ~45%. Adipose tissue lipid-laden foam cells associate in humans with insulin-resistant obesity and could be functionally tied to adipose tissue insulin resistance (Shapiro *et al*. 2013). In addition, the degree of adipose tissue macrophage infiltration in obesity was shown to associate with NAFLD and its severity (Tordjman *et al*. 2009). Our data might suggest a particular causal contribution of ATM lipid content and adipose tissue foam cells to NAFLD, possibly beyond classical inflammatory activation of the ATM, consistent with previous reports (Xu *et al*. 2013, Kratz *et al*. 2014). Alternatively, adipose tissue immune cells other than macrophages (like various subclasses of lymphocytes) may contribute to early reversal of obesity-induced dysregulated adipose tissue endocrine function (Chatzigeorgiou *et al*. 2012).

In summary, this study highlights a previously unrecognized functional contribution of adipose tissue to the metabolic normalization induced by 2 weeks of dietary reversal of obesity. Our results are consistent with the notion that early in obesity reversal, rapid normalization of dysglycemia is matched by a robust improvement in hepatic steatosis and metabolism (though not insulin signaling responsiveness) (Jung *et al*. 2013, Cho *et al*. 2016). These precede normalization of major inflammatory changes induced by obesity in adipose tissue. Nevertheless, adipose tissue’s auto-paracrine and endocrine regulation of lipid handling by macrophages and liver cells is normalized, contributing to the recovery from the metabolic consequences of obesity.

## Supplementary data

Table S1 list of PCR primers and antibodies used

Table S2 Serum lipids and liver enzymes

## Declaration of interest

The authors declare that there is no conflict of interest that could be perceived as prejudicing the impartiality of the research reported.

## Funding

This study was supported in part by grants from the Israel Science Foundation (ISF 874/15) and by the Deutsche Forschungsgemeinschaft (DFG) (SFB 1052/1: Obesity mechanisms (project B2)). M V and S B were supported by the National Institute of Biotechnology in the Negev (NIBN), Ben-Gurion University, Beer-Sheva, Israel. A R is Chair of the Fraida Foundation in Diabetes Research.

## Authors’ contribution statement

M V conducted the study, performed data collection and analysis and data presentation, participated in manuscript writing; S B conducted the study, performed data collection and analysis and data interpretation, participated in manuscript writing; Y H performed data analysis and interpretation; T P participated in data collection and analysis; T T participated in data collection; N S participated in data collection; O N participated in data collection; H S participated in data collection and analysis; A S participated in data collection; A P participated in study design and data analysis; N B participated in data analysis and interpretation; A R participated in study design, data analysis and interpretation and wrote the manuscript.
