# Supplementary material for: Adipose tissue supports normalization of macrophage and liver lipid handling in obesity reversal
Source: J Endocrinol. 2017 Mar 30;233(3):293–305. doi: 10.1530/JOE-17-0007 (PMC5457504; doi:10.1530/JOE-17-0007)
Supplement: Table S1 [file joe-233-293-t001.pdf]

Supplemental Table 1: list of PCR primers and antibodies used

**Taqman system probes for RT-PCR amplification**	
Rplp0(36b4)	Mm01974474_gH
Hprt	Mm00446968_m1
Il6	Mm00446190_m1
Tnfa	Mm00443258_m1
Emr1 (F4/80)	Mm00802529_m1
Itgax (Cd11c)	Mm00498698_m1
Cd163	Mm00474091_m1
Mgl2	Mm00460844_m1
Acaca	Mm01304257_m1
Hmgcr	Mm01282499_m1
Pparg	Mm01184322_m1

**Antibodies for Western Blotting analysis**	
Phospho-Akt (Ser473)	Cell signaling # 4060
Total Akt	Cell signaling # 9272
Phospho-GSK-3α/β (Ser21/9)	Cell signaling # 9331
Total GSK	Cell signaling # 9315
Phospho-SEK1/MKK4 (Ser257/Thr261)	Cell signaling # 9156
Phospho-p38 MAPK (Thr180/Tyr182)	Cell signaling # 4631
Phospho-SAPK/JNK (Thr183/Tyr185)	Cell signaling # 9251
Total SAPK/JNK	Cell signaling # 9252
phosphoTyrosine (PY1000)	Cell signaling # 8954
β-actin	Sigma # A5441
